# Optical Control of Nanomechanical Brownian Motion Eigenfrequencies in Metamaterials

**DOI:** 10.1021/acs.nanolett.1c04900

**Published:** 2022-05-24

**Authors:** Jinxiang Li, Kevin F. MacDonald, Nikolay I. Zheludev

**Affiliations:** † Optoelectronics Research Centre, University of Southampton, Highfield, Southampton SO17 1BJ, United Kingdom; ‡ Centre for Disruptive Photonic Technologies and The Photonics Institute, SPMS, Nanyang Technological University Singapore 637371, Singapore

**Keywords:** photonic metamaterials, thermal motion, nanomechanics, optical control

## Abstract

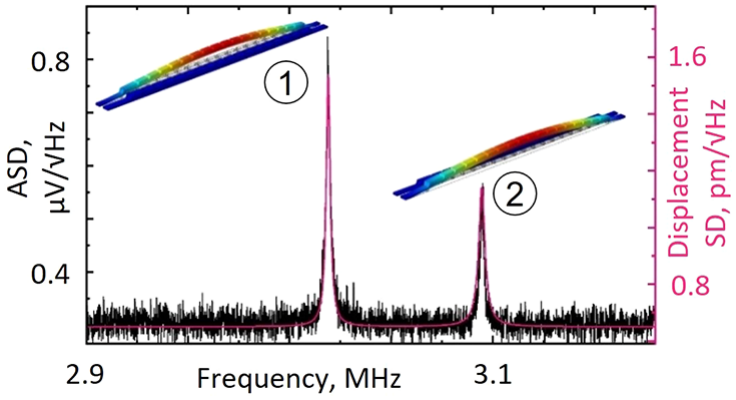

Nanomechanical photonic metamaterials provide a wealth of active switching, nonlinear, and enhanced light-matter interaction functionalities by coupling optically and mechanically resonant subsystems. Thermal (Brownian) motion of the nanostructural components of such metamaterials leads to fluctuations in optical properties, which may manifest as noise, but which also present opportunity to characterize performance and thereby optimize design at the level of individual nanomechanical elements. We show that nanomechanical motion in an all-dielectric metamaterial ensemble of silicon-on-silicon-nitride nanowires can be controlled by light at sub-μW/μm^2^ intensities. Induced changes in nanowire temperature of just a few Kelvin and nonthermal optical forces generated within the structure change the few-MHz Eigenfrequencies and/or picometric displacement amplitudes of motion, and thereby metamaterial transmission. The tuning mechanism can provide active control of frequency response in photonic metadevices and may serve as a basis for bolometric, mass, and micro/nanostructural stress sensing.

By virtue of their low mass and fast (MHz–GHz frequency) response times, nanomechanical oscillators actuated and/or interrogated by light are of fundamental and applied interest in numerous applications, ranging from mass and force sensors to photonic data processing and quantum ground state measurements.^1−13^ As the dimensions of such systems decrease, their thermal (i.e., Brownian) motion assumes increasing importance. By adding noise to induced/controlled movements that underpin the functionality, it can constrain performance, but it also presents opportunity by directly linking observable (far-field optical) properties to geometry, composition, and temperature at the nanoscale. We show here that such motion can be optically controlled at μW/μm^2^ intensities in nanomechanical photonic metamaterials. In an array of mechanically independent and (by design) alternately dissimilar dielectric nanowires, which are at the same time of identical bilayer (i.e., asymmetric) material composition and part of an optically resonant ensemble subject to the fundamental constraint of linear transmission reciprocity, dependences of motion Eigenfrequencies and picometric displacement amplitudes on local light-induced temperature changes can be accurately determined. We further show that the amplitude of high-frequency oscillatory motion driven by nonthermal optical (gradient and radiation pressure) forces is resonantly enhanced, leading to larger changes in transmission, when the nanowires’ natural frequencies are photothermally tuned to coincide with the pump modulation frequency.

In the present study, we employ an all-dielectric metamaterial comprising pairs of dissimilar (by length and width) silicon nanobricks on a free-standing array of flexible silicon nitride nanowires (Figure 1a). It is fabricated on a 200 nm thick Si_3_N_4_ membrane coated by plasma-enhanced chemical vapor deposition with a 115 nm thick layer of amorphous Si. This bilayer is then structured by focused ion beam milling to define rows of alternately short, narrow (720 nm × 210 nm) and long, wide (780 nm × 300 nm) nanobricks in the Si layer on parallel 21 μm long nanowire beams cut through the Si_3_N_4_ layer with a gap size between neighboring nanowires of 170 nm.

**Figure 1:**
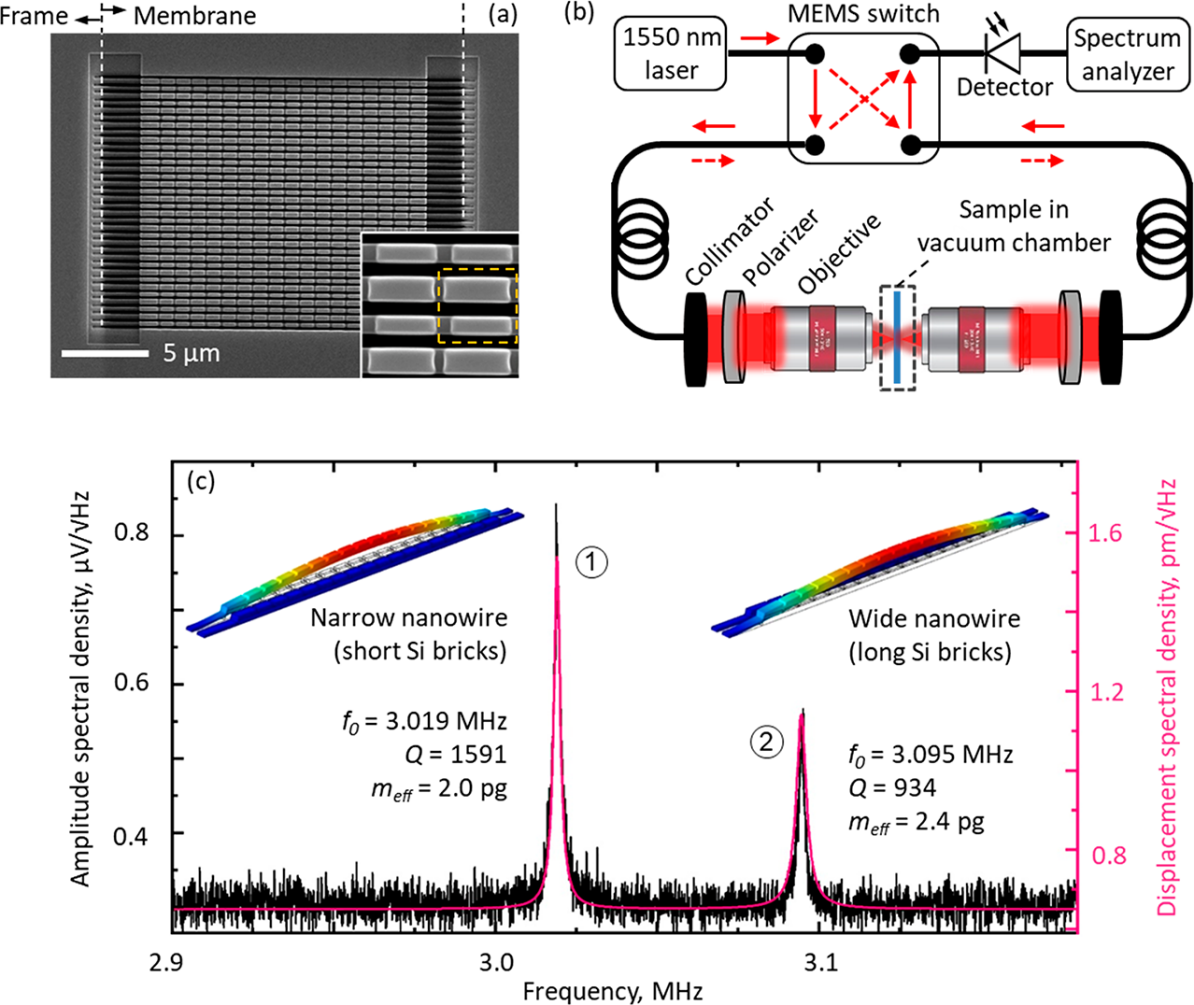
Detecting thermal (Brownian) motion of nanowires within an all-dielectric nanomechanical metamaterial. (a) Scanning electron microscope image of the metamaterial, fabricated on a 21 μm wide free-standing silicon nitride membrane. The inset enlarged section shows detail of the supported silicon nanobricks, and the dashed line denotes a unit cell of the structure. (b) Schematic of experimental apparatus for recording frequency spectra of metamaterial transmission. Other than between the two collimators, light is carried in polarization-maintaining single-mode optical fiber with the MEMS switch providing for inversion of the light propagation direction through the sample. (c) Exemplar measurement of optical transmission amplitude spectral density [for light incident on the silicon nitride side of the sample at a power level of 15.9 μW], showing a pair of peaks associated with the mechanical resonances of two individual nanowires within the array: ①/② a narrower/wider wire decorated with shorter/longer Si bricks. The overlaid magenta curve and calibrated displacement spectral density scale [to the right-hand side] are obtained by fitting eq 1 to the experimental data. Derived values of *f*_0_, *Q*, and *m*_eff_ are shown inset.

The metamaterial structure supports a near-infrared closed mode optical resonance^14^ at a wavelength of 1542 nm (see Supplementary Figure S1), underpinned by the excitation of antiparallel displacement currents in adjacent dissimilar silicon nanobricks by incident light polarized parallel to the long axis of the bricks. In the vicinity of this optical resonance, thermal (Brownian) motion of the nanowires, mutual positional fluctuations of pico- to nanometric amplitude, translate to fluctuations of metamaterial transmission (of order 0.1%) at their few MHz natural mechanical resonance frequencies.^15,16^ These thermomechanical oscillations are detected as peaks in frequency spectra of transmission amplitude spectral density (Figure 1b,c): the metamaterial is mounted in a vacuum chamber at a pressure of 4 × 10^–3^ mbar to exclude air damping of mechanical motion. (It should be noted here that while a classical Brownian particle in a fluid is thermally perturbed by “external” collisions with ambient atoms, the thermal motion of objects under vacuum is driven “internally” by momentum transfer from the annihilation, creation, and interference of phonons.) The sample chamber is located between a confocal pair of 20× (NA 0.4) microscope objectives, via which incident light at a wavelength of 1550 nm is focused onto the sample (to a spot of diameter ∼5 μm at the center of the metamaterial array) and transmitted light is collected. The arrangement includes a fiber-optic MEMS switch to enable transmission measurements in both directions through the sample without disturbance of its position/alignment relative to the beam path. (The “forward” direction of light propagation is designated as that for which light is incident on the Si side of the sample; “backward” is the Si_3_N_4_ side.)

Figure 1c shows a representative measurement of optical transmission amplitude spectral density (ASD) in which peaks associated with the fundamental out-of-plane flexural modes of a pair of individual nanowires, one narrow and one wide decorated, respectively, with short and long Si nanobricks, are seen. (Attribution to this oscillatory mode is confirmed through computational modeling; see Supporting Information.)

Nanowire displacement ASD can be expressed as^17,18^
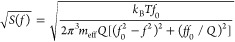
1where *k*_B_ is the Boltzmann constant, *T* is temperature, and for each mode *m*_eff_, *f*_0_, and *Q* are, respectively, the effective mass, natural frequency, and quality factor of the oscillator. Experimental data can thus be calibrated: the vertical scale on the left of Figure 1c converted from signal measured in μV to nanowire displacement in picometres (on the right) by fitting eq 1 to the data. Specifically, we fit a linear superposition of two instances of the expression, one for each of the spectral peaks with co-optimized values of *f*_0_, *Q*, and *m*_eff_. Root mean square (RMS) thermal motion amplitudes can then be evaluated as the square root of an integral of power spectral density (ASD^2^) over frequency. These calculations yield amplitudes of 76 and 67 pm, respectively, for the lower and higher frequency peaks in Figure 1c, which compare extremely well with analytical values of 76 and 68 pm derived from energy equipartition theorem^19^
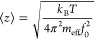
2Figure 2 shows how the Brownian motions characteristics of nanowires, as manifested in the ASD of optical transmission, depend upon (i.e., can be controlled by tuning) incident laser power, and how this dependence differs for the two directions of incident light propagation. With increasing laser power, mechanical Eigenfrequencies redshift (Figure 3a) and RMS displacement amplitudes increase (Figure 3b), both in direct proportion and more rapidly for the backward propagation direction. The behaviors are consistent with a photothermal tuning mechanism, whereby laser-induced heating decreases tensile stress in the nanowires. The effect is more pronounced for the backward direction of light propagation because while forward and backward transmission levels are identical (as they must be in a linear, reciprocal medium) reflectivity and absorption are not (Figure S1).

**Figure 2:**
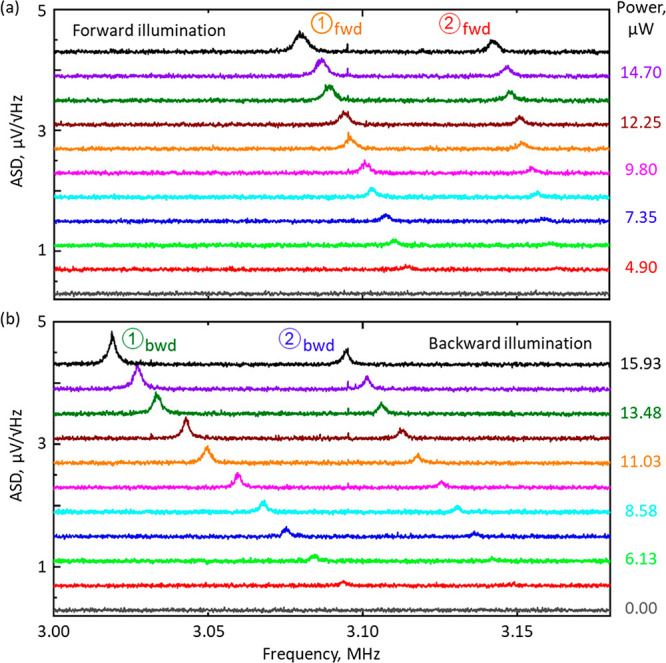
Optical control of nanowire Eigenfrequencies. Transmission amplitude spectral density, showing peaks ① and ② as assigned in Figure 1c, for opposing directions of light propagation through the sample (a) forward and (b) backward [light incident respectively on the silicon and the silicon nitride side], and for a range of laser power levels [as labeled].

**Figure 3:**
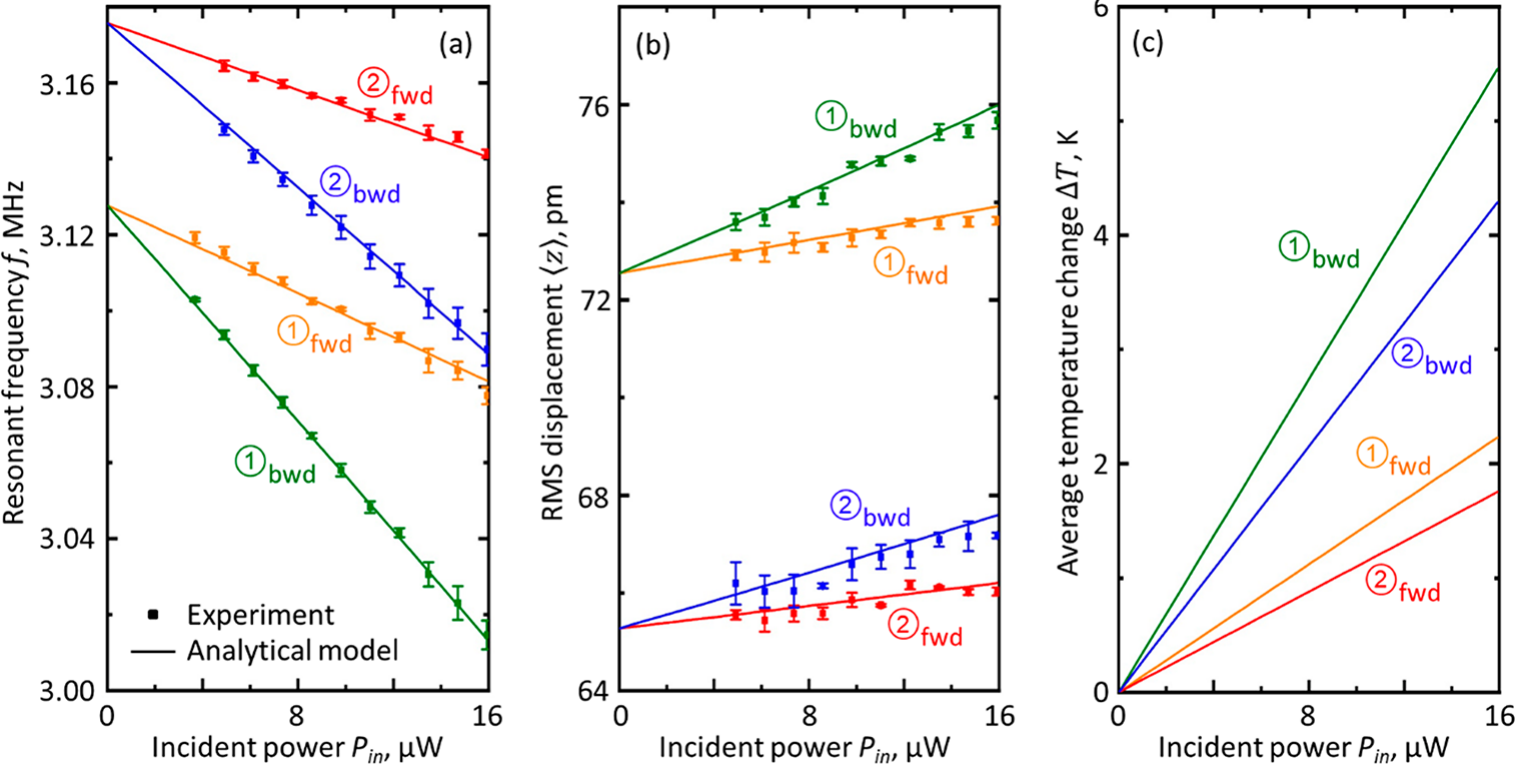
Optical control of nanowire Eigenfrequencies and Brownian motion amplitudes. Dependences for peaks ① and ② in Figure 2 [i.e., for narrow and wide nanowires as identified in Figure 1c, under nominally forward and backward directions of illumination] of (a) resonance frequency, (b) RMS displacement amplitude, and (c) light-induced nanowire temperature change, on incident laser power [total power incident on the metamaterial sample]. Square symbols are experimental data points with error bars given by the standard deviation over three repeated measurement cycles. Solid lines are derived from an analytical description of the photothermal tuning mechanism via a simultaneous best-fit to the four experimental data sets in (a).

An analytical model for optical control of thermomechanical (Brownian) motion resonances, tightly constrained by the requirement to describe the properties of two independent, similar (related) but not identical oscillators - narrow and wide Si_3_N_4_/Si bilayer nanowires, under two similar (related) but not identical regimes of optical excitation - forward and backward directions of illumination, provides for accurate quantitative evaluation of light-induced temperature changes in the individual nanowires and of the corresponding relationships between their resonance frequencies/amplitudes, illumination conditions, and the optical properties of the metamaterial array. From Euler–Bernoulli beam theory,^20^ the stress-dependent fundamental frequency of a doubly clamped beam of homogeneous rectangular cross-section is
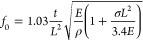
3where *t* and *L* are the thickness and length of the beam, *E* is Youngs’s modulus, ρ is density, and σ is tensile stress along the beam length. The temperature-dependence of stress can be expressed in the form^21^
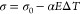
4where *σ*_0_ is the stress at ambient temperature, *ΔT* is the difference between average beam and ambient temperatures, and α is the beam’s thermal expansion coefficient.

Assuming the presence of a heat source uniformly distributed over the rectangular cross-section at the midpoint of the beam, while the two ends are held at ambient temperature (*T*_0_ = 298 K), the equilibrium difference *ΔT* between average beam and ambient temperatures is^21^
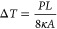
5where κ is thermal conductivity, *A* is the cross-sectional area, and *P* is the power of the heat source.

For the purpose of applying these analytical expressions to the present case, we approximate the silicon nitride nanowires decorated with silicon nanobricks as simple rectangular-section beams of the same length with effective values of *E*, ρ, α, κ, and *A* derived from the material parameters of Si and Si_3_N_4_ and the volume fractions of the two materials in each nanowire type (see Supporting Information). *P* is taken as the optical power absorbed by a nanowire: *P*_abs_ = μγ*P*_in_, where *P*_in_ is the total power incident on the metamaterial, γ is the absorption coefficient of the metamaterial, and μ is the “absorption cross-section” of an individual nanowire.

By substituting eqs 4 and 5 into eq 3 and then fitting that expression simultaneously to all four sets of experimental data points in Figure 3a (see Supporting Information) under constraints that

(i) μ must be identical for forward and backward directions of illumination for a given nanowire (i.e., the same nanowire will intercept the same fraction of incident light in both directions);
(ii) γ must be identical for the two nanowires for a given illumination directions (i.e., as a metamaterial ensemble property, absorption can only have a single value in each direction);
(iii) *σ*_0_ must take a single fixed value (ensuring degeneracy of nominally forward- and backward-illumination zero-power resonant frequencies for each type of nanowire);

we obtain the four solid curves plotted in each panel of Figure 3. The eigenfrequency fitting (Figure 3a) is extremely good and yields zero-power (i.e., ambient temperature) resonant frequencies of 3.18 and 3.13 MHz for the wider and narrower nanowires, respectively. Derived values of μ, 4.9% and 5.6%, respectively, for the narrower and wider nanowires, are consistent to a first approximation with their areas of geometric intersection with the ∼5 μm diameter incident laser spot. However, they are not in proportion simply to the ratio of nanowire widths (0.7:1). This is because the near-field distribution of the electromagnetic field around the metamaterial at resonance is not homogeneous, and the absorption cross section of constituent nanowires is therefore not expected to be directly proportional to its geometric cross-section. Derived values of γ, 10.6% and 26.0%, respectively, for the forward and backward directions of light propagation, correspond well to measured (far-field) values of metamaterial absorption at 1550 nm (Figure S1a) of 13.5% and 23.7%.

Light-induced changes in nanowire temperature (Figure 3c) depend upon the direction of illumination and nanowire dimensions, that is, upon the strength of optical absorption and the rate at which heat is dissipated (the latter being lower for the nanowire of smaller cross-section). The narrow nanowire changes temperature at a rate of 27 K/μW of absorbed power, and the wide nanowire at 19 K/μW. These derived dependences of induced temperature change *ΔT* on laser power map to the theoretical dependences of RMS Brownian motion amplitude presented in Figure 3b via eq 2, using values of *m*_eff_ for the two nanowires established in the above (Figure 1c) calibration of displacement spectral density. The picometrically accurate correlation with experimental data points separately derived from integrals of PSD over frequency is remarkably good.

With the introduction of a second, modulated pump laser, one can observe in probe transmission the interplay between thermomechanical fluctuations and nonthermal optically driven motion, that is, the action of optical forces (Figure 4). Here, we employ a probe laser at a fixed power of 22 μW at a wavelength of 1540 nm, and a pump laser at 1550 nm electro-optically modulated at a fixed frequency of 2.97 MHz. The beams are coincident on the sample in the forward direction (coupled in fiber before the MEMS switch input), and a narrow bandpass filter (at the switch output) ensures that only transmitted probe light reaches the detector. Under these conditions, we observe again in the frequency spectrum of probe transmission peaks relating to Brownian motion at nanowire Eigenfrequencies dependent upon average pump power (i.e., pump-induced temperature change). But also a sharp peak at the pump modulation frequency relating to structural reconfiguration, and thereby probe transmission change, driven by nonthermal optical forces. (There is no coherent temperature oscillation induced by the pump; modulation period is some 2 orders of magnitude shorter than the nanowires’ thermal relaxation time.) The magnitude of this optically induced transmission change is resonantly enhanced by more than an order of magnitude (reaching ∼30%) when the pump modulation frequency coincides with a nanowire’s mechanical eigenfrequency (as shown in the inset in Figure 4).

**Figure 4:**
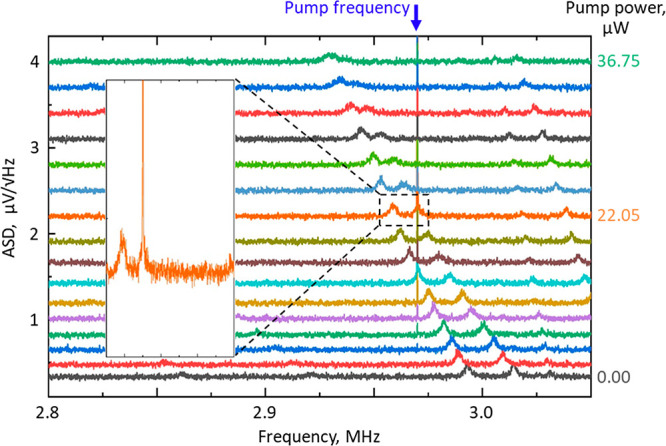
Optical control of nanomechanical motion: thermal and nonthermal mechanisms. Amplitude spectral density of probe transmission (in the forward direction) for a range of pump peak power levels with a fixed pump modulation frequency.

In summary, we have shown that fluctuations in the resonant optical properties of a photonic metamaterial, which are associated with the mechanically resonant Brownian motion of its constituent elements, can be controlled by light at sub-μW/μm^2^ intensities. In an all-dielectric metamaterial ensemble of free-standing silicon nitride nanowires (mechanical oscillators) supporting an array of silicon nanobricks (optical resonators), the few MHz Eigenfrequencies and picometric amplitudes of individual nanowires’ motion are directly proportional to incident laser power, changing, respectively, in consequence of light-induced heating by up to 0.7% and 0.9% per K.

An analytical model for the photothermal tuning mechanism, simply but effectively constrained by the requirements of optical transmission reciprocity in a linear medium, links the local, nanoscopic properties and behaviors of individual nanowires (i.e., at subwavelength scale) to the far-field optical properties of the (micro/macroscopic) metamaterial ensemble. It provides for accurate evaluation of light-induced changes in nanowire temperature and of ambient condition (zero-illumination) Brownian motion Eigenfrequencies and displacement amplitudes.

We further illustrate how nonthermal optical forces generated at the near-IR optical resonance of the metamaterial structure, again at sub-μW/μm^2^ intensities, can be engaged to drive motion at its (photothermally tuned) mechanical Eigenfrequencies and thus to deliver strong light-induced changes in transmission.

The ability to finely tune the nanomechanical resonance characteristics of photonic metamaterials may be beneficial in a variety of metadevice applications where, for example, the frequency of nanostructural oscillation is required to match (or avoid matching) another frequency, such as that of a pulsed laser. The fact that tuning characteristics can (as here) depend strongly upon the direction of light propagation through a metamaterial by simple virtue of bilayer material composition (leading to different levels of reflection and absorption for light incident on opposing sides) may find application in devices to favor/select a single direction of propagation. The accurately quantifiable sensitivity of optical response to nanomechanical properties in such structures also suggests applications to bolometric sensing and detection of changes in mass (e.g., through adsorption/desorption) or micro/nanostructural stress.

## Supporting Information Available

Metamaterial optical properties; Nanowire geometry and mechanical resonance frequencies; Nanowire effective medium parameters; Curve fitting algorithm (PDF)
