# Supplementary material for: Optical Control of Nanomechanical Brownian Motion Eigenfrequencies in Metamaterials
Source: Nano Lett. 2022 May 24;22(11):4301–6. doi: 10.1021/acs.nanolett.1c04900 (PMC9185736; doi:10.1021/acs.nanolett.1c04900)
Supplement: Supplementary file 1 — nl1c04900_si_001.pdf [file nl1c04900_si_001.pdf]

Optical Control of Nanomechanical Brownian Motion Eigenfrequencies in Metamaterials

Jinxiang Li[*], Kevin F MacDonald[*‡], and Nikolay I. Zheludev[* †]

** Optoelectronics Research Centre, University of Southampton, Highfield, Southampton, SO17 1BJ, UK*

† Centre for Disruptive Photonic Technologies & The Photonics Institute, SPMS, Nanyang Technological University Singapore, 637371, Singapore

‡ Email: kfm@orc.soton.ac.uk

Metamaterial optical properties

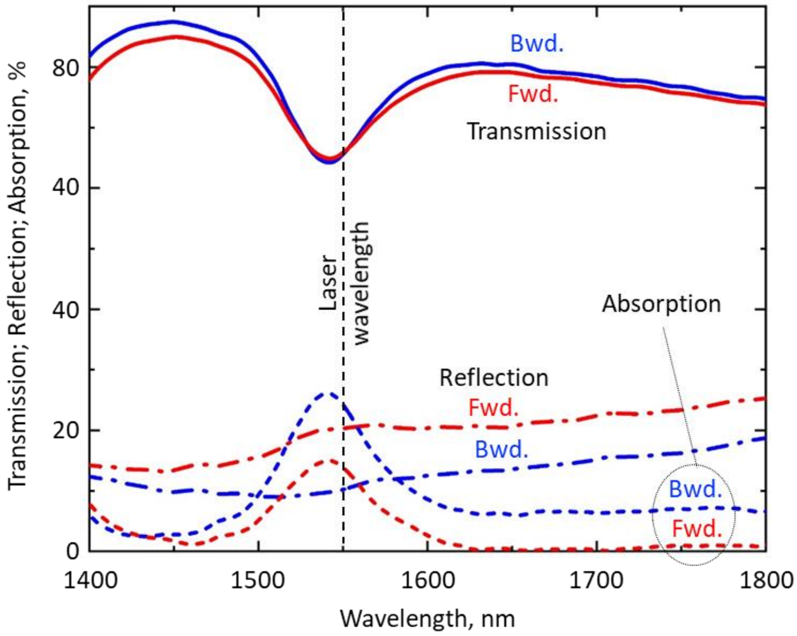

Fig. S1: Spectral dispersion of metamaterial transmission *T* and reflection *R* measured using a microspectrophotometer, and absorption *A* calculated as [100-(*T*+*R*)], for incident light polarized parallel to the nanowires. Spectra are measured for the two different directions of light propagation (nominally forward and backward) through the sample by removing it from the instrument, turning it over, and reinserting it. Resulting positional alignment imperfections account for the small discrepancies between the two transmission spectra (which would be identical in an ideally reciprocal pair of measurements).

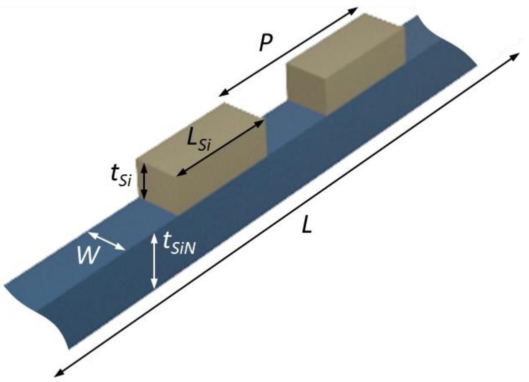

	Nanowire type ①	Nanowire type ②
Nanowire length L, µm	21.0	
Width W, nm	210	300
Si_3N_4 thickness t_{SiN}, nm	200	
Si thickness t_{Si}, nm	115	
Si brick length L_{Si}, nm	720	780
Si brick period p, nm	910	

Nanowire geometry and mechanical resonance frequencies

The metamaterial is fabricated on a 185 nm thick, Si_3N_4 membrane coated with 100 nm of amorphous Si, structured to define rows of alternately narrow (type ①) and wide (type ②) nanowires each supporting a row of eighteen short and long, respectively, Si nano-bricks, according to the dimensional schematic in Fig. S2.

Nanowire mechanical properties are simulated using the structural mechanics module in COMSOL Multiphysics (finite element method). For the ideal rectilinear geometry of Fig. S2, and densities and Young's moduli of silicon nitride and silicon as in Table S1 below, Eigenfrequencies of the fundamental out-of-plane flexural modes are estimated as 3.04 and 3.13 MHz respectively for the type ① (narrower) and type ② (wider) nanowires.

These are sufficiently close to each other and to the measured frequencies (Fig. 1c) as to confirm that the Brownian motion peaks observed in experiment are associated with this mode of oscillation – discrepancies being accounted for by manufacturing imperfections and internal stress within the silicon nitride. (For comparison, Eigenfrequencies of the fundamental in-plane flexural modes are much higher, at around 4.5 MHz.)

Table S1: Material properties

Fig. S2: Dimensional schematic of a section of an all-dielectric nanomechanical metamaterial nanowire element

	**Si**	**Si_3N_4**
**Density ρ, kg.m^{-3}**	2330	3100
**Young's Modulus E, GPa**	165	260
**Thermal expansion coefficient α, K^{-1}**	1.0×10^{-6}	2.8×10^{-6}
**Thermal conductivity κ, Wm^{-1}K^{-1}**	1.5	2.0

Nanowire effective medium parameters

For the purpose of analytically modeling the photothermal tuning of Brownian motion resonances, the nanowires are assumed to be of simple rectangular cross-section and homogenous material composition. The real value is taken for length L and physical properties are assigned effective values (Table S2) based upon the volume fractions of Si and Si_3N_4 present, according to the expression:

$$X_e = \frac{X_{Si}V_{Si} + X_{SiN}V_{SiN}}{V_{Si} + V_{SiN}}$$

where X is density ρ, Young's modulus E, the thermal expansion coefficient α, or thermal conductivity κ (values of X_{SiN} and X_{Si} being given in Table S1). V_{SiN} and V_{Si} are respectively the volumes of silicon and silicon nitride, evaluated from electron microscopic measurements to encompass real-sample deviations from the ideal geometry of Fig. S2: specifically, the fact that the Si nano-bricks at each end of the row on each nanowire are of slightly different length, and that the sections of bare silicon nitride at each end, between the nano-brick array and the anchor points, are over-milled to a reduced thickness of 80 nm. Effective cross-sectional area A_e is then evaluated as $(V_{SiN} + V_{Si})/L$.

Table S2: Nanowire effective medium parameters

	**Nanowire type ①**	**Nanowire type ②**
**Density ρ_e, kg.m^{-3}**	2870	2859
**Young's Modulus E_e, GPa**	232	230
**Thermal expansion coefficient α_e, K^{-1}**	2.263×10^{-6}	2.237×10^{-6}
**Thermal conductivity κ_e, Wm^{-1}K^{-1}**	1.851	1.844
**Cross-sectional area A_e, nm^2**	52760	76945

Curve fitting algorithm

By substituting Eqs. (4) and (5) into Eq. (3) [given on pages 6-7 of the manuscript] one obtains the following expression for the dependence of nanowire Eigenfrequency f on incident laser power P_{in}:

$$f(P_{in}) = 1.03\frac{t}{L^2}\sqrt{\frac{E}{\rho}\left[1 + \frac{\left(\sigma_0 - \alpha E\frac{\mu\gamma P_{in}L}{8\kappa A}\right)L^2}{3.4E}\right]}$$

We employ a nonlinear least-squares solver ('lsqcurvefit' in MATLAB) to fit four iterations of this equation to the four f-against-P_{in} datasets in Fig. 3a – i.e. for type ① and ② nanowires under forward and backward illumination. Parameters in blue are fixed to the effective values specified in Table S2, according to nanowire type, while L assumes a fixed value of 21.0 μm. The solver then iteratively seeks convergence, with a step tolerance of 1×10^{-6}, on two values of μ (for type ① and ② nanowires regardless to illumination direction) and γ (for forward and

backward illumination regardless to nanowire type), and a single value of σ_0, using effective thickness t as a free fitting parameter (i.e. to encompass imperfect knowledge of nanostructural geometry and material parameters, e.g. deviations from ideal rectilinear shapes, differences between real and data book values of Si and SiN parameters, etc.). It aims to minimize the value of

$$\sum_{i=1}^{n}\left(f(P_{in}^{i}) - F(P_{in}^{i})\right)^2$$

where $F(P_{in}^{i})$ is the experimentally measured Eigenfrequency at input power P_{in}^{i}.

The quality of fitting is evaluated via a coefficient of determination:

$$R^2 = 1 - \frac{\sum_{i=1}^{n}\left(f(P_{in}^{i}) - F(P_{in}^{i})\right)^2}{\sum_{i=1}^{n}\left(F(i) - \overline{F(i)}\right)^2}$$

where $\overline{F(\mathrm{i})} = \frac{\sum_{i=1}^{n} F(i)}{n}$ is the mean experimentally observed frequency.

In the present case, we achieve a value of $R^2 = 0.998$ for derived values of $\mu_{①} = 4.9\%$ and $\mu_{②} = 5.6\%$, $\gamma_{FWD} = 10.6\%$ and $\gamma_{BWD} = 26.0\%$, and $\sigma_0 = 5.6$ MPa, with effective thicknesses of 139 and 142 nm respectively for type ① and ② nanowires.
